# Genome Sequence of *Micromonospora* sp. NBS 11-29, an Antibiotic and Hydrolytic Enzyme Producer, Isolated from River Sediment in Brazil

**DOI:** 10.1128/genomeA.00552-17

**Published:** 2017-07-13

**Authors:** Simone Ichiwaki, Aline C. M. M. Costa, Eliane G. Silva, Lina R. M. Rada, Felipe R. Lima, Mabel P. Ortíz-Vera, Leandro M. Garrido, Maria Inês Z. Sato, Welington L. Araújo, Gabriel Padilla

**Affiliations:** a NAP-BIOP, Department of Microbiology, Institute of Biomedical Sciences, University of São Paulo, São Paulo, Brazil; b Department of Environmental Analysis, Environmental Company of São Paulo State (CETESB), São Paulo, São Paulo, Brazil

## Abstract

The genus *Micromonospora* comprises actinomycetes with high biotechnological potential, due to their ability to produce secondary metabolites and enzymes. In this study, we report the draft genome sequence of *Micromonospora* sp. NBS 11-29, which showed antibacterial, cellulolytic, and xylanolytic activities under *in vitro* conditions.

## GENOME ANNOUNCEMENT

The genus *Micromonospora* belongs to the family *Micromonosporaceae*, which includes aerobic and filamentous Gram-positive bacteria with micellar development and single-spore sporangiophores (1). The colonies are typically orange, turning red, brown, or black on sporulation (2). These bacteria have been reported in a wide range of environments, such as in soil, freshwater or marine habitats, root nodules of legumes, and actinorhizal plants, as well as in extreme environments, such as Antarctic sandstone (3–7). *Micromonospora* spp. are best known as sources of hydrolytic enzymes and important secondary metabolites with pharmacological activities, such as oligosaccharide antibiotics, antitumor anthraquinones and anthracyclines (8–10), and also as plant growth promoters (3, 11). *Micromonospora* sp. NBS 11-29 was isolated from sediment of Promissão Reservoir (23°37′08″ N, 47°23′22″ W), which belongs to the Tietê River Basin in São Paulo State, Brazil. *Micromonospora* sp. NBS 11-29 is able to degrade complex polysaccharides, such as carboxymethycellulose, pectin, and xylan under *in vitro* conditions, and demonstrated antibiotic activity against pathogenic microorganisms, such as *Candida albicans* and *Staphylococcus aureus*.

The genomic DNA of *Micromonospora* sp. NBS 11-29 was extracted from 7-day-old culture using the Wizard Genomic DNA purification kit (Promega) and sequenced with the Illumina HiSeq 2000 platform using 100-bp paired-end reads, to reach a 350-fold depth of coverage. The assembly of the reads was performed with the SOAPdenovo2 method (12), and the raw data were annotated with the software tool Prokka (13). The featured prediction tools used by Prokka included Prodigal (for coding DNA sequences), RNAmmer (for rRNAs), Aragorn (for tRNA genes), SignalP (for signal leader peptides), and Infernal (for non-coding RNA).

The draft of the *Micromonospora* sp. NBS 11-29 genome has 6,473,937 bp and a G+C content of 71.57%. The annotation analysis predicted 6,036 genes, of which 5,971 are coding DNA sequences, 63 are tRNAs, and 2 are tmRNAs. The prediction of the gene clusters was performed with the antiSMASH pipeline (14), which predicted biosynthetic gene clusters that code for bioactive secondary metabolites, including 4 terpenes, 10 type I polyketide synthases (PKS I), 1 siderophore, 1 lasso peptide, and 11 gene clusters predicted to encode hybrid molecules, such as a terpene-bacteriocin hybrid and an NRPS-PKS I-oligosaccharide-Aminocoumarin hybrid.

Our data indicate that *Micromonospora* sp. NBS 11-29 has genes related to the production of potentially interesting secondary metabolites and hydrolytic enzymes, suggesting its high biotechnological potential as a bioactive-molecules producer.

### Accession number(s).

This whole-genome shotgun project has been deposited in GenBank under the accession number NAPR00000000. The version described in this paper is the first version, NAPR01000000.
