# Correction for Ichiwaki et al., “Genome Sequence of *Micromonospora* sp. NBS 11-29, an Antibiotic and Hydrolytic Enzyme Producer, Isolated from River Sediment in Brazil”

**DOI:** 10.1128/MRA.01346-18

**Published:** 2018-10-18

**Authors:** Simone Ichiwaki, Aline C. M. M. Costa, Eliane G. Silva, Lina R. M. Rada, Felipe R. Lima, Mabel P. Ortíz-Vera, Leandro M. Garrido, Maria Inês Z. Sato, Welington L. Araújo, Gabriel Padilla

**Affiliations:** a NAP-BIOP, Department of Microbiology, Institute of Biomedical Sciences, University of São Paulo, São Paulo, Brazil; b Department of Environmental Analysis, Environmental Company of São Paulo State (CETESB), São Paulo, Brazil

## AUTHOR CORRECTION

Volume 5, no. 28, e00552-17, 2017, https://doi.org/10.1128/genomeA.00552-17. Page 2: The first sentence of Acknowledgments should read as follows. “This project was supported by the Pró-Reitoria de Pesquisa/University of São Paulo (USP), CNPq (grant 02458/2013-3), CAPES (institutional student grants), and FAPESP (grant 2017/12647-0).”

